# The study on the effect of mercury pollution on soil microorganisms around mercury mining area

**DOI:** 10.1038/s41598-023-48932-6

**Published:** 2023-12-07

**Authors:** Jianxiong Du, Yuxiang Ren, Jianfeng Li, Shuqing Zhang, Huiqiong Huang, Jie Liu

**Affiliations:** 1 School of Management Science and Engineering, Guizhou University of Finance and Economics, Guiyang, 550025 China; 2 School of Foreign Languages, Guizhou Normal University, Guiyang, 550025 China; 3 Institute of Soil and Environment Bioremediation in Karst Habitats, Key Laboratory of Biological Resources Exploitation and Utilization in Universities of Guizhou Province, Guizhou Normal College, Guiyang, 550018 China; 4 College of Public Management, Guizhou University of Finance and Economics, Guiyang, 550025 China

**Keywords:** Microbiology, Ecology, Environmental sciences

## Abstract

In order to further explore the effects of soil mercury pollution on soil microbial diversity and community structure, soil samples were randomly collected from 2 m, 20 m, 30 m, 500 m and 650 m periphery of Wanshan mining area, as 5 different treatments. Each treatment had 4 replicates. Soil microbial DNA was extracted from 20 soil samples, and then high-throughput sequencing technology was used to analyse the structure and distribution of bacterial and fungal communities. The results showed that the number of bacterial and fungal communities in T0–T30 treatments was significantly larger than that in T500–T650 treatments at order, family and genus level. Whatever, the number of uniquely distributed bacterial and fungal communities among 4 replicates soil samples was quite different at order, family and genus level. The results of the effect on the microbial community structure showed that there were both the same dominant bacterial and fungal communities, and the different dominant bacterial and fungal communities at any classification level, moreover, the number of same dominant bacterial and fungal communities was larger than that of different dominant bacterial and fungal communities. The results of relationship between soil environment factors and bacterial and fungal community structure showed that distance (Hg^2+^), EC and pH had a high correlation with community structure, especially the distance factor, that is, the content of mercury in soil had the highest effects on community structure. The internal heterogeneity of soil caused significant differences in bacterial and fungal community structure, and the emergence of dominant bacterial and fungal communities was a manifestation of better adaptability to long-term mercury stress and other stresses in soil, which will provide a scientific reference for further exploring the mechanism of mercury enrichment between microorganisms and plants.

## Introduction

Soil is an important link between water and atmosphere for material circulation. Soil carries about 90% of pollutants in the environment. Mercury was one of the main control pollutants in 129 priority control pollutants^1–3^. Mercury in soil was often enriched and transferred through crops, and then enriched and transferred to animals and humans through various food chains, resulting in serious ecological problems^4,5^. Soil bacteria, fungi and actinomycetes are the main components of the microflora in the soil ecosystem. When the soil is subjected to heavy metal stress, the soil microflora can reflect the change of soil environmental quality, so it can be used as one of the important biological indicators, and bacteria were more sensitive to heavy metal pollution than fungi^6^. In recent year, there have been many reports on the effects of mercury pollution on plants, and some plants with strong resistance to mercury stress have been found^7,8^, but there were few reports on the effects of mercury pollution on soil microorganisms^9,10^. Harris–Hellal and coworkers showed that when the mercury concentration was low (0–1 mg/kg), the microbial communities in the forest soil did not change significantly. When the mercury concentration increased to 20 mg/kg, the diversity and genetic structure of the soil microbial communities changed significantly^11^. The combined pollution of heavy metals cadmium and mercury could reduce the diversity of soil microbial communities, and the abundance ratio of gram-positive bacteria to gram-negative bacteria increases with the increase of pollution concentration^12,13^. Mercury in soil inhibited the growth of fungi, actinomycetes and bacteria, and seriously affected soil bacterial activity and bacterial community structure. In high-concentration mercury-contaminated environments, *gemmatimonadetes* were still active but *Nitrospirae* was declining. Fungi was prone to resistance in high-concentration mercury-contaminated environments^14,15^. Frossard and coworkers found that when the mercury concentration reached 32 mg/kg, it seriously affected the community structure, composition and diversity of bacteria and fungi in forest soil^16^. In order to further explore the effect of mercury on soil microorganisms, this experiment was carried out to collected soil samples at different distances from the abandoned mercury mining area in Wanshan, Tongren, Guizhou Province, China. Meanwhile, high-throughput sequencing was used to explore the effects of different concentrations of mercury in soils at different distances from the mercury mining area on soil microbial diversity and community structure, which provided theoretical support for further exploration of the synergistic mercury enrichment mechanism of mercury-resistant bacteria and plants.

## Methods

### General situation of soil sample plot

**Figure 1:**
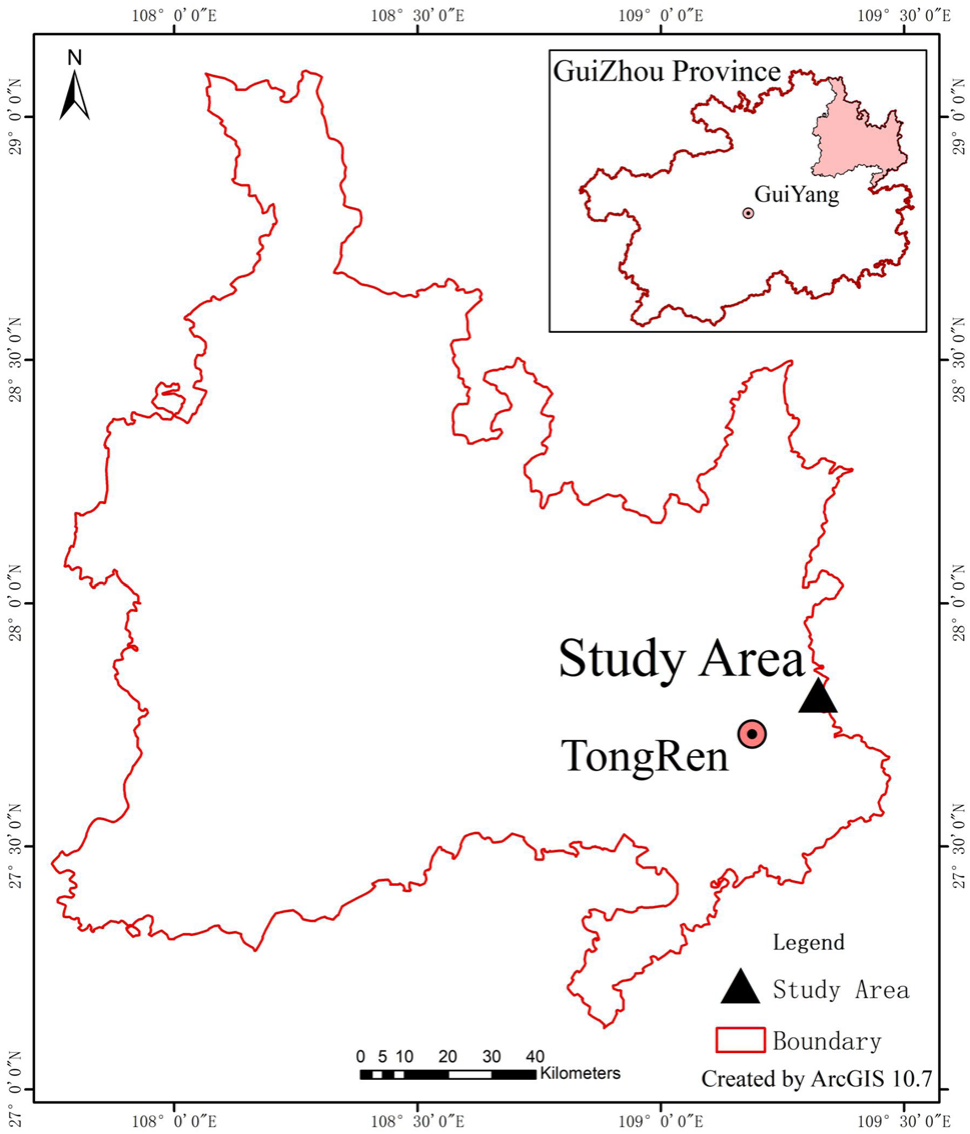
Location map of study area.

Wanshan mercury areas (E:109°07′–109°24′; N: 27°24′–27°38′) is located in the east of Tongren City, Guizhou Province, China (Fig. 1). It is 26 km wide from east to west and 22 km long from north to south. The total area of the region is 842 km^2^, and the mining area accounts for 45 km^2^. The terrain of the whole territory is low in the east and high in the west, and the central uplift, from the middle. The part tilts to the southeast on three sides. The altitude is 260–997 m, with an average of 850 m. It belongs to subtropical humid monsoon climate. The annual average temperature is 13.7 °C, the maximum temperature is 34.6 °C, and the minimum temperature is − 10.4 °C. Summer, The season is warm and rainy, and the precipitation is 1379 mm. Since the excavation of cinnabar in the Qin and Han Dynasties, the history of mercury mining and smelting in Wanshan has been more than two thousand years. In 2001, Wanshan Mercury Mine in Guizhou was closed due to resource depletion. The map of study area was created by ArcGIS software (Version 10.7 USA).

### Sample collection

Five sampling points were selected at different distances around the abandoned area of Wanshan Mercury Mine in Tongren, Guizhou Province, China. Soil samples were collected at 16:44 p.m. on May 9, 2022, in summer. The T0 treatment soil samples, numbered MSO1, MSO2, MSO3 and MSO4, were collected at different points about 2 m from the sewage outlet of the mercury mining area. The T20 treatment soil samples, numbered CMSO21, CMSO22, CMSO23, and CMSO24 were collected from the wasteland of *perennial ryegrass* about 20 m away from the mining area. The T30 treatment soil samples, numbered GMSO301, GMSO302, GMSO303, and GMSO304 were collected from the wasteland of *perennial ryegrass* about 30 m away from the mining area. The T500 treatment soil samples, numbered GMSO500, GMSO501, GMSO502, and GMSO503 were collected from new cultivated land at different points about 500 m away from the mercury mining area. The T650 treatment soil samples, numbered GMSO651, GMSO652, GMSO653 and GMSO654 were collected from long-term cultivated land at different points about 650 m away from the mercury mining area, each treatment had 4 replicates.

All soil samples were taken from 0 to 20 cm soil under the surface, removed impurities, crushed, and sieved by 1 mm. A total of 20 soil samples were tested. In order to facilitate statistical analysis, all 20 soil samples were grouped. MSO1, MSO2, MSO3, MSO4, CMSO21, CMSO22, CMSO23, CMSO24, GMSO301, GMSO302, GMSO303 and GMSO304 soil samples were in the range of 2–30 m from the sewage outlet of the mercury mining area, and these soil samples were classified into the proximal group (CR). Similarly, GMSO500, GMSO501, GMSO502, GMSO503, GMSO651, GMSO652, GMSO653 and GMSO654 soil samples were collected 500–650 m away from the sewage outlet of the mercury mining area, and these soil samples were classified into the distal group (FBR). Two sets of soil samples were prepared, one for the determination of soil physical and chemical properties, and another for the determination of soil microbial DNA.

### Mercury measurement

At first, 0.1–0.2 g over 200 mesh of dry soil was weighed accurately and placed in a 50 mL centrifuge tube, 5 mL deionized water was added, 5 mL freshly prepared aqua regia was added again, after being shaken well, and being heated in a 95 °C water bath in a fume hood 5 min, 1 mL BrCl was added to continue water bath for 30 min, after being cooled, being set the volume to 50 mL. Being placed for more than 24 h, BrCl oxidizes various forms of mercury to Hg^2+^, 0.4 mL hydroxylamine hydrochloride was added 30 min before the determination to remove the migration, an appropriate volume of supernatant was taken in a 10 mL borosilicate glass reaction flask, 0.4 mL SnCl_2_ was added, Determination of mercury by cold atomic absorption mercury analyzer (F732-V,China)^3^, The detection limit of the instrument was 0.05 μg/L.

### The basic physical and chemical properties of the tested soils

The basic physical and chemical properties of soil samples were shown in Table 1. The content of basic nutrients in the soil samples was determined according to the standard analysis method^17^. In all 20 soil samples, pH was from 6.5 to 8.5; the content of available N was from 7.94 to 9.11 mg/kg, the mean of available N was T0 > T650 > T30 > T500 > T20; the content of available P was from 10.13 to 12.14 mg/kg, the mean of available P was T650 > T30 > T0 > T500 > T20; the content of available K was from 27.24 to 30.14 mg/kg, the mean of available P was T30 > T0 > T500 > T650 > T20; the content of Hg^2+^ was from 45.42 mg/kg to 167.14 mg/kg, the mean of Hg^2+^ was T20 > T0 > T650 > T30 > T500, Hg^2+^ content of T0 treatment and T20 treatment was higher than that of T30, T500, and T650 treatment (*P* < 0.05), Hg^2+^ content of T650 treatment was higher than that of T30 and T500 treatment (*P* < 0.05).

**Table 1:** Physiochemical properties of the tested soils.

Soil number		Distance from the mercury mining mouth(m)	Available N (mg/kg)		Available P (mg/kg)		Available K(mg/kg)		EC (us/cm)	pH	Hg^2+^(mg/kg)	
T0	MSO1	0.13	9.06	9.04 ± 0.06a	11.14	11.06 ± 0.07b	29.21	29.24 ± 1.21a	122	7.5	147.04	140.45 ± 15.56a
	MSO2	0.16	9.11		11.02		30.06		124	7.5	139.23	
	MSO3	0.18	8.98		11.08		30.14		125	7.5	119.51	
	MSO4	0.22	9.02		10.99		27.54		123	7.5	156.03	
T20	CMSO1	21.2	8.07	8.03 ± 0.07c	10.34	10.43 ± 0.41c	27.24	27.70 ± 0.49a	112	8.0	167.14	152.44 ± 22.22a
	CMSO2	20.10	8.01		10.13		27.31		114	8.0	139.25	
	CMSO3	19.60	7.94		10.22		28.07		110	8.0	128.33	
	CMSO4	20.32	8.11		11.04		28.16		113	8.5	175.02	
T30	GMSO301	30.50	8.97	8.63 ± 0.46ab	12.03	11.69 ± 0.45a	30.04	29.29 ± 1.16a	119	7.5	69.15	63.48 ± 11.18c
	GMSO301	30.20	9.07		12.10		30.12		122	8.0	72.31	
	GMSO301	29.80	8.31		11.43		29.36		121	7.5	47.29	
	GMSO301	30.10	8.16		11.19		27.62		126	7.5	65.18	
T500	GMSO500	500.20	8.09	8.37 ± 0.44bc	10.21	10.67 ± 0.47bc	30.02	29.16 ± 1.03a	114	8.5	61.51	59.77 ± 11.34c
	GMSO501	500	8.21		10.32		28.22		123	8.5	45.42	
	GMSO502	500.10	8.15		11.03		28.31		116	8.5	73.06	
	GMSO503	499.80	9.02		11.11		30.07		126	8.5	59.10	
T650	GMSO651	650.30	9.11	8.88 ± 0.34ab	11.39	11.90 ± 0.34a	29.11	28.57 ± 0.80a	123	6.5	96.32	109.44 ± 12.35b
	GMSO652	650.20	8.98		12.01		28.52		120	7.0	112.04	
	GMSO653	650.40	9.05		12.14		29.19		126	6.5	125.27	
	GMSO654	650.10	8.37		12.06		27.45		124	7.0	104.13	

Data are means土standard deviation. The different case letters indicate that the means are significantly different among soils (*P* < 0.05) with Duncan test.

### DNA extraction

Total community genomic DNA extraction was performed using a E.Z.N.A™ Mag-Bind Soil DNA Kit (Omega,M5635-02,USA), following the manufacturer's instructions. The concentration of the DNA was measured by using a Qubit 4.0 (Thermo,USA) to ensure that adequate amounts of high-quality genomic DNA had been extracted.

### 16S rRNA gene amplification by PCR

Our target was the V3-V4 hypervariable region of the bacterial 16S rRNA gene. PCR was started immediately after the DNA was extracted. The 16S rRNA V3-V4 amplicon was amplified using 2 × Hieff^®^ Robust PCR Master Mix (Yeasen, 10105ES03, China). Two universal bacterial 16S rRNA gene amplicon PCR primers (PAGE purified) were used: the amplicon PCR forward primer (CCTACGGGNGGCWGCAG) and amplicon PCR reverse primer (GACTACHVGGGTATCTAATCC). The reaction was set up as follows: microbial DNA(10 ng/μl) 2 μl; amplicon PCR forward primer (10 μM)1 μl; amplicon PCR reverse primer (10 μM)1 μl; 2xHieff^®^ Robust PCR Master Mix (Yeasen,10105ES03, China)(total 30 μl). The plate was sealed and PCR performed in a thermal instrument (Applied Biosystems9700, USA) using the following program: 1 cycle of denaturing at 95 °C for 3 min, first 5 cycles of denaturing at 95 °C for 30 s, annealing at 45 °C for 30 s, elongation at 72 °C for 30 s, then 20 cycles of denaturing at 95 °C for 30 s, annealing at 55 °C for 30 s, elongation at 72 °C for 30 s and a final extension at 72 °C for 5 min. The PCR products were checked using electrophoresis in 2% (w/v) agarose gels in TBE buffer (Tris,boricacid,EDTA) stained with ethidium bromide (EB) and visualized under UV light.

### ITS rRNA gene amplification by PCR

Our target was the ITS1-ITS2 hypervariable region of the fungal ITS rRNA gene. PCR was started immediately after the DNA was extracted. The ITS rRNA ITS1-ITS2 amplicon was amplified using 2 × Hieff^®^ Robust PCR Master Mix (Yeasen, 10105ES03, China). Two universal fungal ITS rRNA gene amplicon PCR primers (PAGE purified) were used: the amplicon PCR forward primer (CTTGGTCATTTAGAGGAAGTAA) and amplicon PCR reverse primer (GCTGCGTTCTTCATCGATGC). The reaction was set up as follows: microbial DNA(10 ng/μl) 2 μl; amplicon PCR forward primer (10 μM)1 μl; amplicon PCR reverse primer (10 μM)1 μl; 2xHieff® Robust PCR Master Mix (Yeasen, 10105ES03, China) (total 30 μl). The plate was sealed and PCR performed in a thermal instrument (Applied Biosystems9700, USA) using the following program: 1 cycle of denaturing at 95 °C for 3 min, first 5 cycles of denaturing at 95 °C for 30 s, annealing at 45 °C for 30 s, elongation at 72 °C for 30 s, then 20 cycles of denaturing at 95 °C for 30 s, annealing at 55 °C for 30 s, elongation at 72 °C for 30 s and a final extension at 72 °C for 5 min. The PCR products were checked using electrophoresis in 2% (w/v) agarose gels in TBE buffer (Tris, boricacid, EDTA) stained with ethidium bromide (EB) and visualized under UV light.

### 16S, ITS gene library preparation, quantification, and sequencing

Hieff NGSTM DNA Selection Beads (Yeasen, 10105ES03, China) were used to purify the free primers and primer dimer species in the amplicon product. Samples were delivered to Sangon BioTech (shanghai) for library preparation using universal lllumina adaptor and index. Before sequencing, the DNA concentration of each PCR product was determined using a Qubit^®^ 4.0 Green double-stranded DNA assay and it was quality controlled using a bioanalyzer (Agilent 2100, USA). Depending on coverage needs, all libraries can be pooled for one run. The amplicons from each reaction mixture were pooled in equimolar ratios based on their concentration. Sequencing was performed using the lllumina MiSeq system (lllumina MiSeq, USA), according to the manufacturer's instructions.

### Sequence processing, OTU clustering, Representative tags alignment and Biological classification

After sequencing, The two short lllumina readings were assembled by PEAR software (version 0.9.8) according to the overlap and fastq files were processed to generate individual fasta and qual files, which could then be analyzed by standard methods. The effective tags were clustered into operational taxonomic units (OTUs) of ≥ 97% similarity using Usearch software (version 11.0.667). Chimeric sequences and singleton OTUs (with only one read) were removed, after which the remaining sequences were sorted into each sample based on the OTUs. The tag sequence with the highest abundance was selected as a representative sequence within each cluster. Bacterial and fungal OTU representative sequences were classified taxonomically by blasting against the RDP Database and UNITE fungal ITS Database, respectively.

## Results

### The distribution of bacterial and fungal communities at the order level

**Figure 2:**
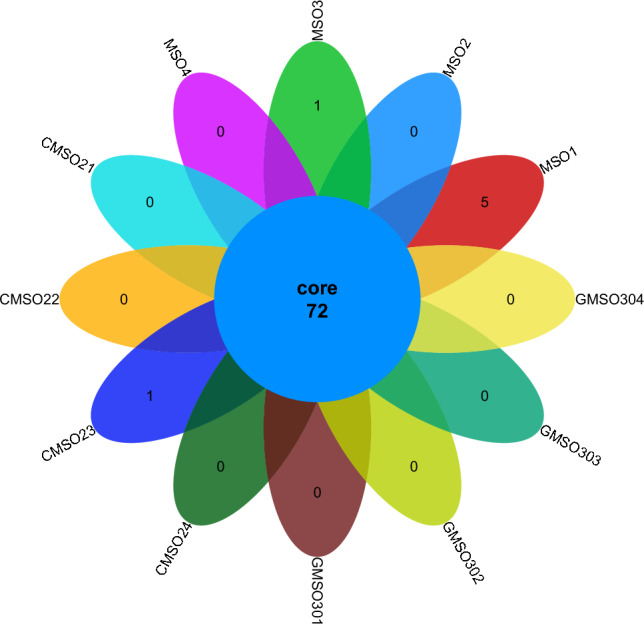
The distribution of bacterial communities in 12 soil samples of the proximal group (CR) at the order level.

**Figure 3:**
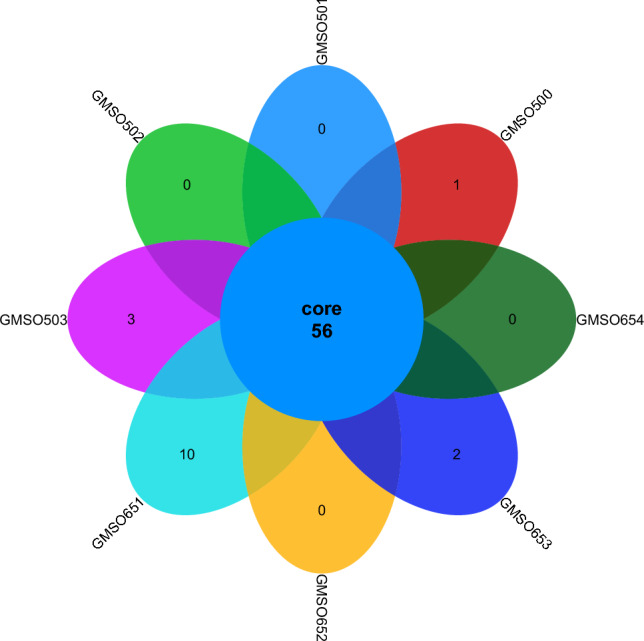
The distribution of bacterial communities in 8 soil samples of the distal group (FBR) at the order level.

At the order level, the distribution of bacterial communities in the 12 soil samples of the proximal group (CR) was shown in Fig. 2. Among them, the number of co-distributed bacterial communities in 12 soil samples was 72, and the number of uniquely distributed bacterial communities in the 12 soil samples was very different. Among them, the number of uniquely distributed bacterial communities in MSO1 was 5, only 1 in MSO3 and CMSO23 soil samples, no uniquely distributed bacterial communities in the other 9 soil samples. The bacterial community distribution of 8 soil samples in the distal group (FBR) was shown in Fig. 3. The number of co-distributed bacterial communities in the 8 soil samples was 56, of which 10 uniquely distributed bacterial communities in GMSO651 soil samples, 3 uniquely distributed bacterial communities in GMSO503 soil samples, 2 in GMSO653 soil samples, and 1 in GMSO500 soil samples. The results of bacterial community distribution of all 20 soil samples showed that the number of bacterial communities in each soil sample of 12 soil samples in the proximal group (CR) was about 72, and in each soil sample of 8 soil samples in the distal treatment group (FBR) was about 58. The number of bacterial communities in T0, T20, and T30 treatments was larger than that in T500 and T650 treatments.

**Figure 4:**
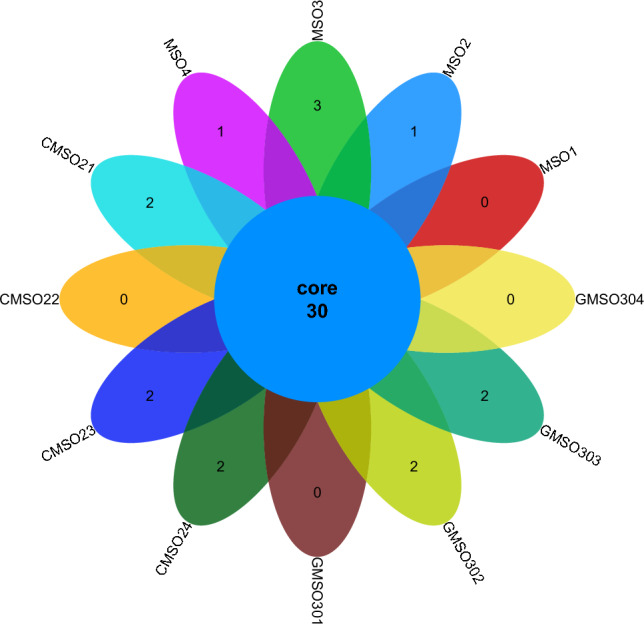
The distribution of fungal communities in 12 soil samples of the proximal group (CR) at the order level.

**Figure 5:**
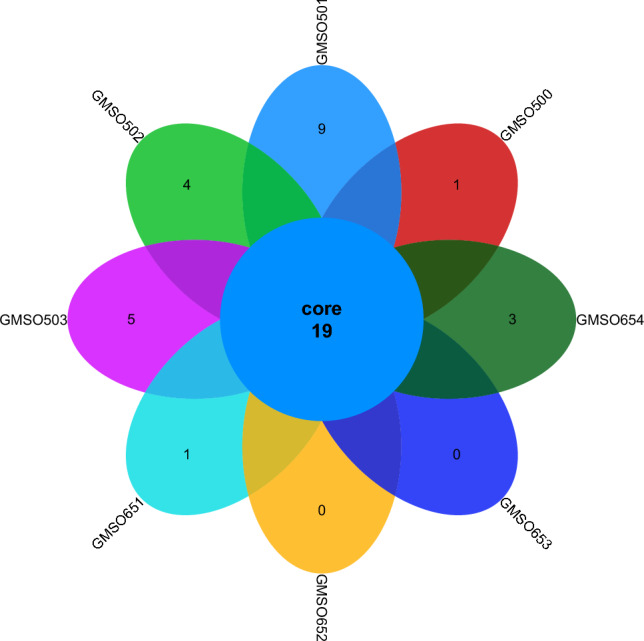
The distribution of fungal communities in 8 soil samples of the distal group (FBR) at the order level.

At the order level, the distribution of fungal communities in 12 soil samples of the proximal group (CR) was shown in Fig. 4. The total number of fungal communities in all 12 soil samples was 30, but the number of uniquely distributed fungal communities to each of the 12 soil samples was very different. Among them, the number of uniquely distributed fungal communities in MSO3 soil sample was 3, 2 uniquely distributed fungal communities in CMSO21, CMSO23, CMSO24, GMSO302, and GMSO303 soil samples, 1 uniquely distributed fungal communities in MSO2 and MSO4 soil samples, and 0 uniquely distributed fungal communities in MSO1, CMSO22, GMSO301, and GMSO304 soil samples. The distribution of fungal communities in 8 soil samples of the distal group (FBR) was shown in Fig. 5. The number of co-distributed fungi in 8 soil samples was 19, of which 9 uniquely distributed fungal communities in GMSO501 soil samples, 5 uniquely distributed fungal communities in GMSO503 soil sample, 3 uniquely distributed fungal communities in GMSO654 soil sample, 1 uniquely distributed fungal communities in GMSO500 and GMSO651 soil samples, and 0 uniquely distributed fungal communities in GMSO652 and GMSO653 soil samples. From the analysis of the number of fungal communities in all 20 soil samples, the number of fungal communities per sample in the 12 soil samples was about 31, and the number of fungal communities per soil sample in the 8 soil samples was about 22. The number of fungal communities in T0, T20, and T30 treatments was larger than that in T500 and T650 treatments. At the order level, the total number of bacterial communities in both the proximal group and the distal group was larger than that of fungal communities.

### The distribution of soil bacterial and fungal communities at family level

**Figure 6:**
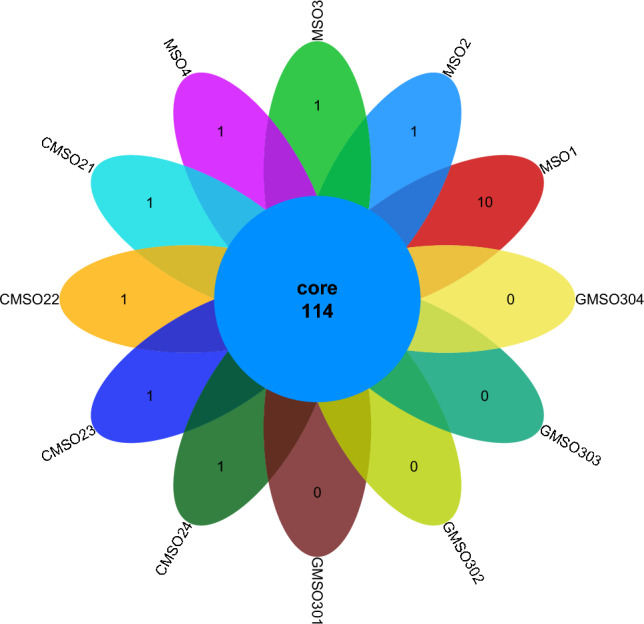
The distribution of bacterial communities in 12 soil samples of the proximal group (CR) at the family level.

**Figure 7:**
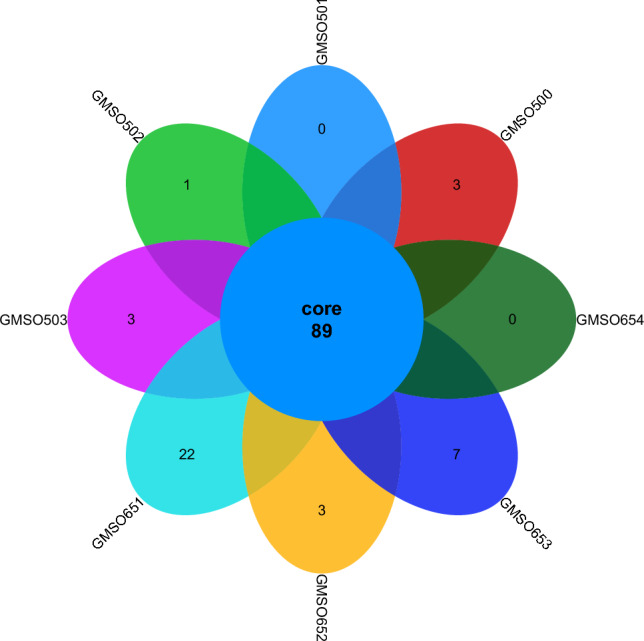
The distribution of bacterial communities in 8 soil samples of the distal group (FBR) at the family level.

**Figure 8:**
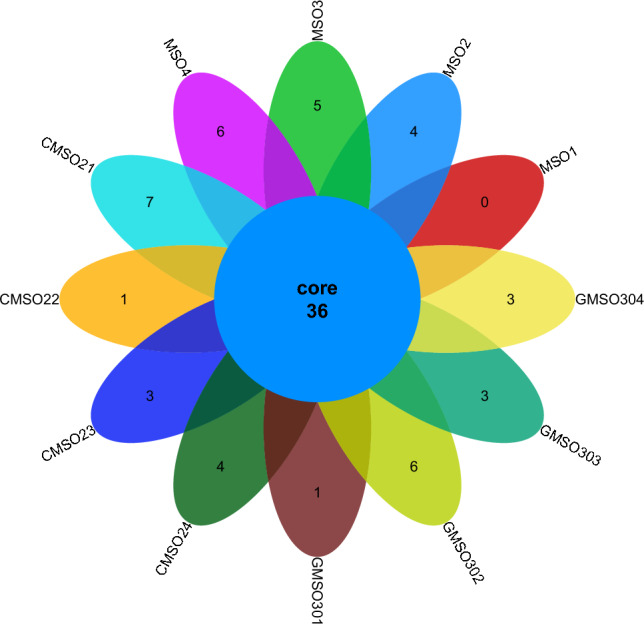
The distribution of fungal communities in 12 soil samples of the proximal group (CR) at the family level.

**Figure 9:**
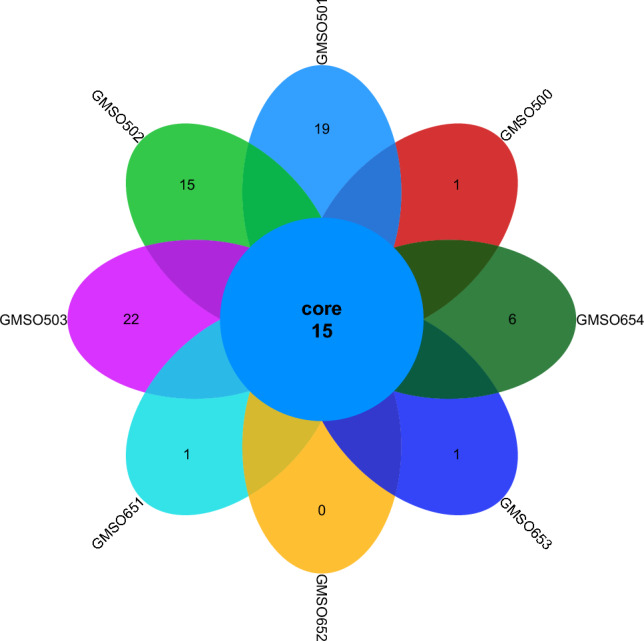
The distribution of fungal communities in 8 soil samples of the distal group (FBR) at the family level.

At the family level, the distribution of bacterial communities in 12 soil samples in the proximal group (CR) was shown in Fig. 6. The total number of bacterial communities in 12 soil samples was 114, and the number of uniquely distributed bacterial communities to each of the 12 soil samples was quite different. Among them, the number of uniquely distributed bacterial communities in MSO1 soil sample was 10. 1 uniquely distributed bacterial communities in MSO2, MSO3, MSO4, CMSO21, CMSO22, CMSO23 and CMSO24 soil samples, while 0 uniquely distributed bacterial communities in GMSO301, GMSO302, GMSO303 and GMSO304 soil samples. The distribution of bacterial communities in 8 soil samples of the distal group (FBR) was shown in Fig. 7. There were 89 bacterial communities in 8 soil samples, and the number of uniquely distributed bacterial communities in 8 soil samples was quite different. Among them, the number of uniquely distributed bacterial communities in GMSO651 soil sample was 22, 7 uniquely distributed bacterial communities in GMSO653 soil sample, 3 uniquely distributed bacterial communities in GMSO500, GMSO503 and GMSO652 soil samples, 1 uniquely distributed bacterial communities in GMSO502 soil sample, 0 uniquely distributed bacterial communities in GMSO501 and GMSO654 soil samples. From the analysis of the number of bacterial communities in all 20 soil samples, the number of bacterial communities in each sample of 12 soil samples was about 115, and the number of bacterial communities in each sample of 8 soil samples was about 92. The number of bacterial communities in T0, T20, and T30 treatments was larger than that in T500 and T650 treatments. At the family level, the distribution of fungal communities in 12 soil samples of the proximal group (CR) was shown in Fig. 8. In the proximal group (CR), the number of co-distributed fungal communities in 12 soil samples was 36, and the number of uniquely distributed fungal communities in 12 soil samples was quite different. Among them, the number of uniquely distributed fungal communities in CMSO21 soil sample was 7, 6 uniquely distributed fungal communities in MSO4 and GMSO302 soil samples, 5 uniquely distributed fungal communities in MSO3 soil sample, 4 uniquely distributed fungal communities in MSO2 and CMSO24 soil samples, 3 uniquely distributed fungal communities in GMSO303, GMSO304, and CMSO23 soil samples. 1 uniquely distributed fungal communities in CMSO22 and GMSO301 soil samples, while 0 uniquely distributed fungal communities in MSO1soil sample. The distribution of fungal communities in 8 soil samples of the distal group (FBR) was shown in Fig. 9. The number of co-distributed fungal communities in 8 soil samples was 16, and the number of uniquely distributed fungal communities among the 8 soil samples was quite different. Among them, the number of uniquely distributed fungal communities in GMSO503, GMSO501 and GMSO502 soil samples was 22, 19 and 15 respectively, while 1 uniquely distributed fungal communities in GMSO500 soil sample. Among GMSO651, GMSO652, GMSO653 and GMSO654 soil samples, only GMSO654 soil sample had 6, GMSO651 and GMSO653 soil samples had 1 respectively, and GMSO652 soil sample had 0. From the distribution of fungal communities in all 20 soil samples, the number of fungal communities in each sample of 12 soil samples was about 39, and the number of fungal communities in each sample of 8 soil samples was about 22. The number of fungal communities distribution in T0, T20, and T30 treatments was larger than that in T500 and T650 treatments at the family level.

### The distribution of bacterial and fungal communities at genus level

**Figure 10:**
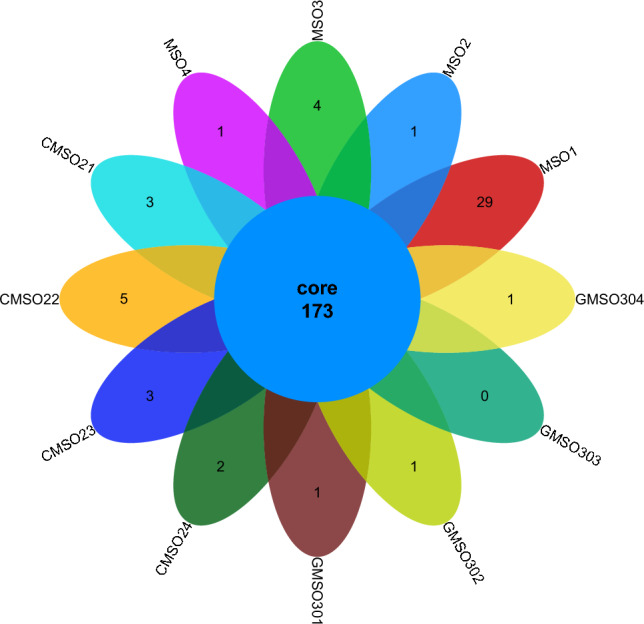
The distribution of bacterial communities in 12 soil samples of the proximal group (CR) at the genus level.

**Figure 11:**
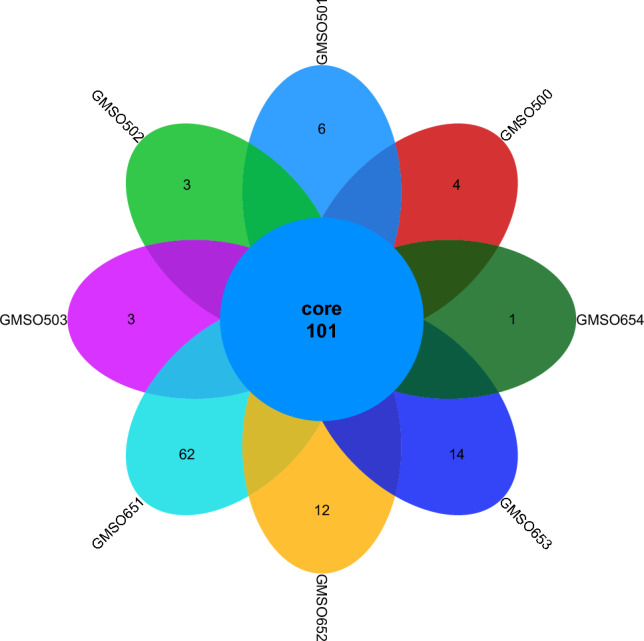
The distribution of bacterial communities in 8 soil samples of the distal group (FBR) at the genus level.

At the genus level, the bacterial community distribution of 12 soil samples in the proximal group (CR) was shown in Fig. 10. The number of co-distributed bacterial communities in 12 soil samples was 173, and the number of uniquely distributed bacterial communities to each of the 12 soil samples was very different. Among them, the number of uniquely distributed bacterial communities in MSO1 soil sample was the largest, reaching 29. 1, 4, and 1 uniquely distributed bacterial communities in MSO2, MSO3, and MSO4 soil samples respectively. 3, 5, 3, and 2 uniquely distributed bacterial communities in CMSO21, CMSO22, CMSO23, and CMSO24 soil samples respectively. 1, 1, 0, 1 uniquely distributed bacterial communities in GMSO301, GMSO302, GMSO303 and GMSO304 soil samples respectively. The distribution of bacterial communities of 8 soil samples in the distal (FBR) group was shown in Fig. 11. The number of co-distributed bacterial communities in 8 soil samples was 101, and the number of uniquely distributed bacterial communities to each soil sample varied greatly. Among them, the number of uniquely distributed bacterial communities in GMSO651 soil samples was 62, and 12, 14, 1 uniquely distributed bacterial communities in GMSO652, GMSO653, and GMSO654 soil samples respectively. 4, 6, 3 and 3 uniquely distributed bacterial communities in GMSO500, GMSO501, GMSO502 and GMSO503 soil samples respectively. According to the distribution of bacterial communities in all 20 soil samples, the number of bacterial community distribution in each sample of 12 soil samples was about 177, while the number of bacterial communities in each sample of 8 soil samples was about 114, the number of bacterial communities in T0, T20, and T30 treatments was larger than that in T500 and T650 treatments.

**Figure 12:**
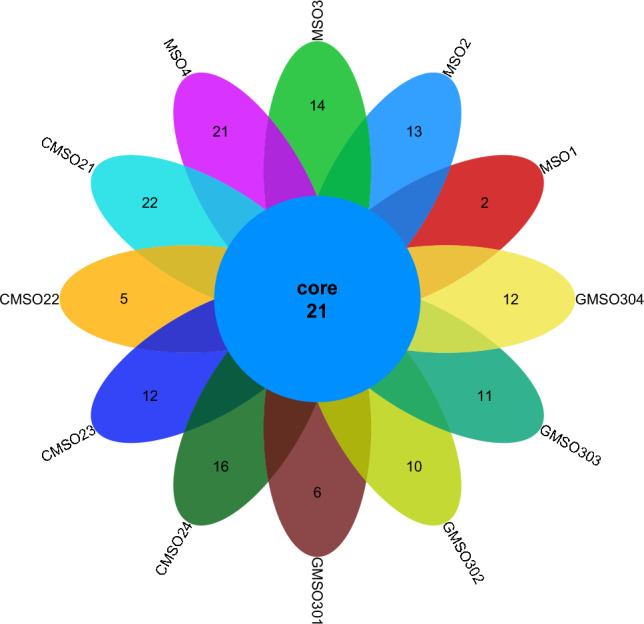
The distribution of fungal communities in 12 soil samples of the proximal group (CR) at the genus level.

**Figure 13:**
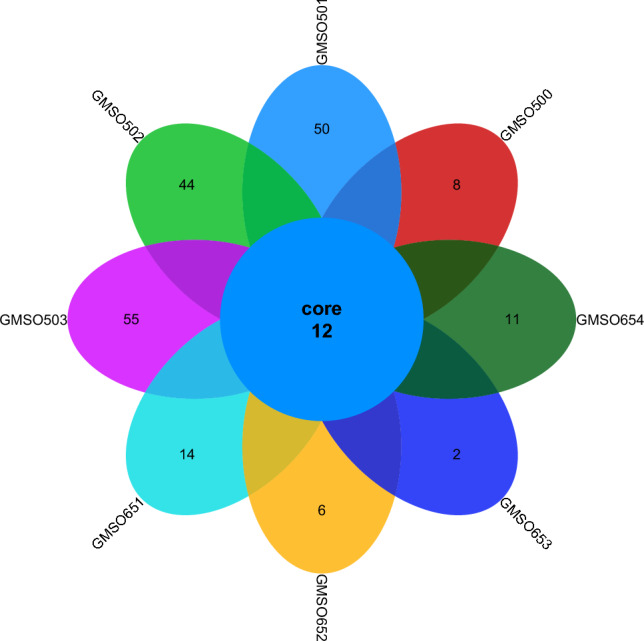
The distribution of fungal communities in 8 soil samples of the distal group (FBR) at the genus level.

At the genus level, the distribution of fungal communities in 12 soil samples of the proximal group (CR) was shown in Fig. 12. The number of co-distributed fungal communities in 12 soil samples was 21, but there was a big difference in the number of uniquely distributed fungal communities among the 12 soil samples. The number of uniquely distributed fungal communities in MSO1, MSO2, MSO3 and MSO4 soil samples was 2, 13, 14, 21 respectively. 22, 5, 12, 16 uniquely distributed fungal communities in CMSO21, CMSO22, CMSO23 and CMSO24 soil samples respectively. 6, 10, 11, 12 uniquely distributed fungal communities in GMSO301, GMSO302, GMSO303 and GMSO304 soil samples respectively. The distribution of fungal communities in 8 soil samples of the distal group (FBR) was shown in Fig. 13. The total number of fungal communities in the 8 soil samples was 12, but the number of uniquely distributed fungal communities among the 8 soil samples varied greatly. Among them, the number of uniquely distributed fungal communities in GMSO500, GMSO501, GMSO502, GMSO503 soil samples was 8, 50, 44, 55, respectively, and 14, 6, 2, 11 uniquely distributed fungal communities in GMSO651, GMSO652, GMSO653, GMSO654 soil samples respectively. From the distribution of fungal communities in all 20 soil samples, the number of fungal communities each sample in 12 soil samples was about 33, and the number of fungal communities per sample in the 8 soil samples was about 35, The number of fungal communities in T0, T20, and T30 treatments was close to the number of fungal communities in T500 and T650 treatments.

### Alpha diversity analysis

The alpha diversity indices of bacterial communities in 5 treatments were shown in the Table 2. The number of reads in T0–T650 treatments was 46,321, 42,898, 42,062, 44,585, 57,825, i.e. T650 > T0 > T500 > T20 > T300; the number of OUTs in T0–T650 treatments was 2558, 2267, 2088, 2151, 1541, i.e. T0 > T20 > T500 > T30 > T650; Shannon indices of T0–T650 treatments were 6.30911975, 6.256060, 6.0918122, 5.9032615, 5.103864 respectively, that is T0 > T20 > T30 > T500 > T650; Simpson indices of T0–T650 treatments were 0.00851625, 0.00630375, 0.0071275, 0.0122905, 0.0453070 respectively, that is T0 < T30 < T20 < T500 < T650. Comprehensive judgment based on the results of above 4 indices, the alpha diversity indices of bacterial communities in 5 treatments was T0 > T20 > T30 > T500 > T650.

**Table 2:** Statistical table of bacterial communities alpha diversity.

Soil sample		Reads	OTUs	Shannon Index		Simpson Index	
T0	MSO1	35,846	2473	6.5521	6.30911975	0.003989	0.00851625
	MSO2	48,222	2699	6.356606		0.009506	
	MSO3	47,730	2290	5.742979		0.015878	
	MSO4	53,487	2772	6.584794		0.004692	
T20	CMSO1	43,283	2379	6.303647	6.256060	0.005523	0.00630375
	CMSO2	44,785	2106	6.18422		0.005933	
	CMSO3	46,269	2390	6.37952		0.004639	
	CMSO4	37,257	2195	6.156853		0.00912	
T30	GMSO301	45,797	2014	5.90724	6.0918122	0.008004	0.0071275
	GMSO302	40,009	2038	6.078248		0.007725	
	GMSO303	42,766	2279	6.247333		0.006103	
	GMSO304	39,678	2022	6.134428		0.006678	
T500	GMSO500	31,575	2106	6.138057	5.9032615	0.006991	0.0122905
	GMSO501	59,912	2375	6.065342		0.007856	
	GMSO502	40,239	1861	5.369534		0.026926	
	GMSO503	46,614	2265	6.040113		0.007389	
T650	GMSO651	64,768	2317	6.070957	5.103864	0.01084	0.0453070
	GMSO652	53,616	697	4.383491		0.043944	
	GMSO653	60,428	1475	4.49185		0.103022	
	GMSO654	52,489	1678	5.469158		0.023422	

The alpha diversity indices of fungal communities in 5 treatments were shown in Table 3. The number of reads in T0–T650 treatments was 69,062, 50,849, 89,767, 62,446, 51,248, i.e. T30 > T0 > T500 > T650 > T20; the number of OUTs in T0–T650 treatments was 559, 313, 610, 509, 159, i.e. T30 > T0 > T500 > T20 > T650; Shannon indices of T0–T650 treatments were 2.42374775, 2.63471725, 2.4174655, 3.260999, 4.02980475 respectively, i.e. T650 > T500 > T20 > T0 > T30, Simpson indices of T0–T650 treatments were 0.3465155, 0.2690235, 0.2884785, 0.1538875, 0.05412475 respectively, i.e. T650 < T500 < T20 < T30 < T0. Comprehensive judgment based on the results of above 4 indices, The alpha diversity indices of fungal communities in 5 treatments was T650 > T500 > T30 > T20 > T0.

**Table 3:** Statistical table of fungal alpha diversity.

Soil sample		Reads	OTUs	Shannon Index		Simpson Index	
T0	MSO1	45,621	191	1.098074	2.42374775	0.659097	0.3465155
	MSO2	80,213	620	2.727059		0.267726	
	MSO3	78,827	683	2.872671		0.22027	
	MSO4	71,590	744	2.997187		0.238969	
T20	CMSO1	30,492	460	3.857819	2.63471725	0.084499	0.2690235
	CMSO2	64,132	163	1.332677		0.592299	
	CMSO3	56,055	318	2.519291		0.202738	
	CMSO4	52,720	312	2.829082		0.196558	
T30	GMSO301	99,736	609	2.577139	2.4174655	0.183603	0.2884785
	GMSO301	73,065	570	2.567143		0.251882	
	GMSO301	81,577	647	1.781741		0.530917	
	GMSO301	104,691	614	2.743839		0.187512	
T500	GMSO500	39,561	259	3.611093	3.260999	0.063412	0.1538875
	GMSO501	44,095	443	3.608671		0.111481	
	GMSO502	94,691	622	3.121362		0.129372	
	GMSO503	71,437	713	2.70287		0.311261	
T650	GMSO651	55,617	264	4.767795	4.02980475	0.01524	0.05412475
	GMSO652	58,200	74	3.877419		0.033157	
	GMSO653	38,506	95	4.158947		0.021239	
	GMSO654	52,669	205	3.315058		0.146863	

### Beta diversity analysis

**Figure 14:**
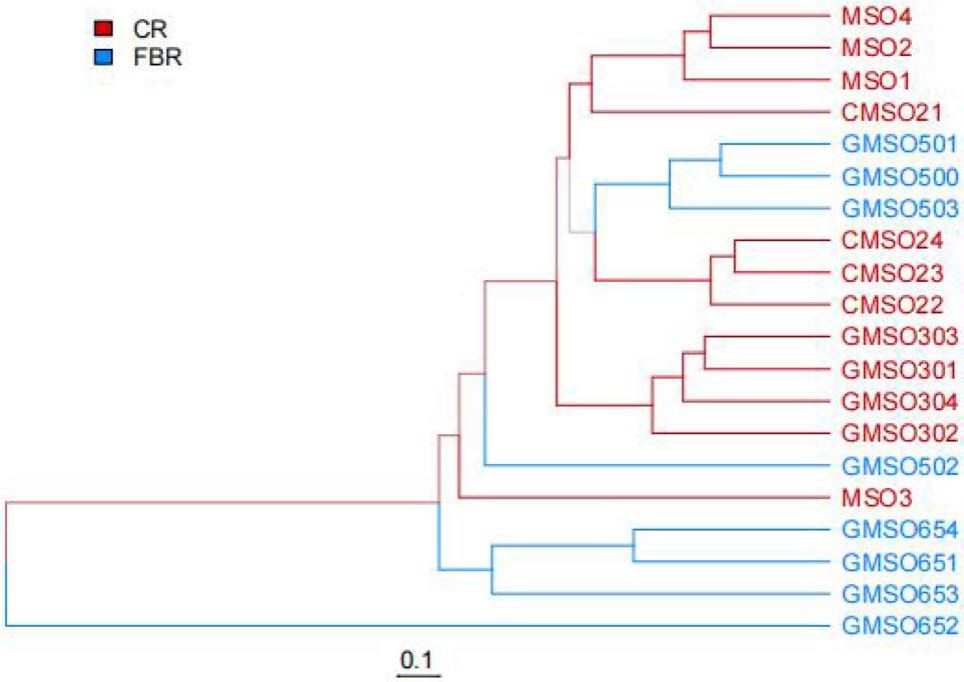
Weighted unifrac tree anaylsis of bacteria at OTU level.

The beta diversity analysis of bacterial communities among 20 soil samples was shown in Fig. 14 at the OTU level. From the bacterial community structure of 20 soil samples in T0–T650 treatments, except for the MSO3, CMS021, GMSO502 soil samples, the bacterial community composition of the other 18 soil samples was nearer to that of the soil samples in their respective treatment. Based on the similarity of bacterial communities of soil samples among T0–T650 treatments, from the perspective of phylogenetic distance, the results of the occurrence of bacterial communities in 5 treatments of soil samples from near to far was T20, T0, T500, T30, T650. By comparing the similarity of bacterial communities between CR and FBR group soil samples, the results showed that bacterial communities of T650 treatment in FBR group soil samples had lower similarity with T0, T20, T30 treatments in CR groups soil samples, but bacterial communities of T500 treatment in FBR group soil samples had higher similarity with T0, T20, T30 treatments in CR groups soil samples.

**Figure 15:**
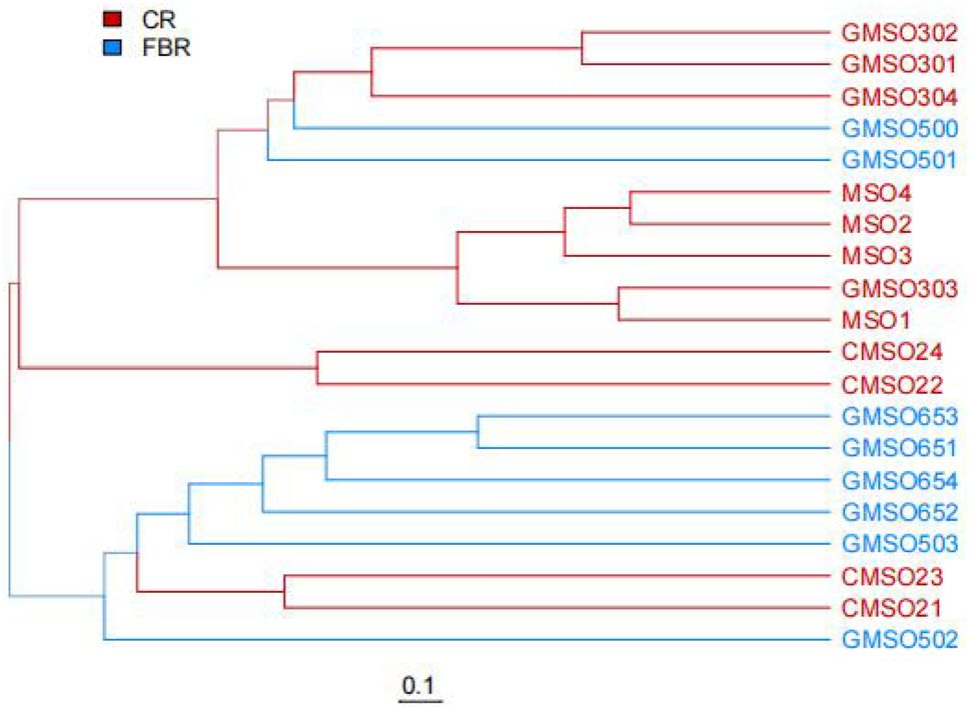
Weighted unifrac tree anaylsis of fungi at OTU level.

The beta diversity analysis of fungal communities among 20 soil samples was shown in Fig. 15 at the OTU level. From the fungal community structure of 20 soil samples in T0–T650 treatments, except for the GMSO502 and GMSO503 soil samples, the fungal community composition of the other 18 samples was nearer to that of the soil samples in their respective treatments. Based on the similarity of fungal community structure of soil samples among T0–T650 treatments, From the perspective of phylogenetic distance, the results of the occurrence of fungal communities in 5 treatments of soil samples from near to far was T0, T30, T20, T650, T500. By comparing the similarity of fungal communities between CR and FBR group soil samples, the results showed that fungal communities of T500 and T650 treatments in FBR group soil samples had lower similarity with T0 and T30 treatments in CR groups soil samples.

### The community structure of bacteria and fungi at the order level

**Figure 16:**
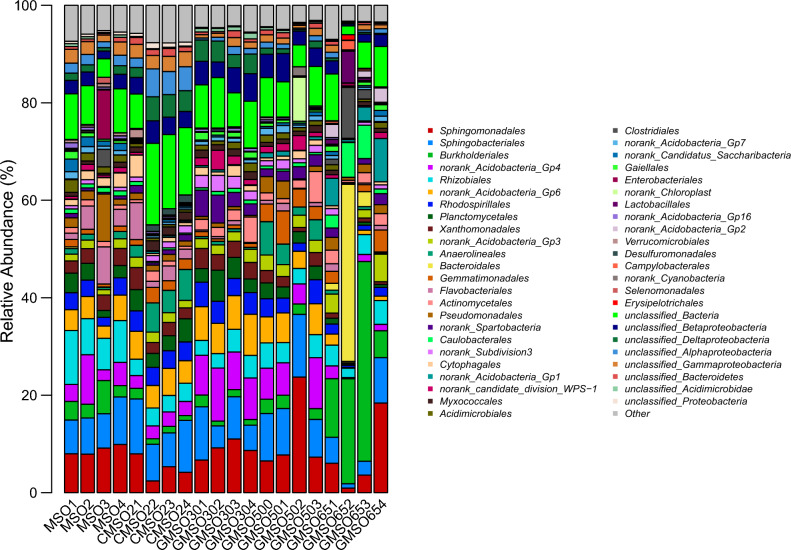
The relative abundance of bacterial communities in 20 soil samples at the order level.

At the order level, the relative abundance of bacterial communities in 20 soil samples was shown in Fig. 16. *Sphingomonadales*(8.74%), *Rhizobiales*(8.38%), *Sphingobacteriales*(7.78%), *Gp4*(norank_*Acidobacteria*_Gp4)(5.16%) had higher average relative abundance in T0 treatment; *Sphingobacteriales*(9.10%), *Gp6*(norank_*Acidobacteria*_Gp6)(5.14%), *Sphingomonadales*(4.96%) in T20 treatment; *Sphingomonadales*(8.88%), *Gp4*(norank_*Acidobacteria*_Gp4)(8.83%), *Sphingobacteriales*(7.33%), *Gp6*(norank_*Acidobacteria*_Gp6)(7.12%) in T30 treatment; *Sphingomonadales*(11.31%), *Sphingobacteriales*(9.97%), *Gp4*(norank_*Acidobacteria*_Gp4)(6.90%), *Gp6*(norank_*Acidobacteria*_Gp6)(5.32%) in T500 treatment; *Burkholderiales*(20.01%), *Bacteroidales*(10.27%), *Sphingomonadales*(7.24%), *Sphingobacteriales*(4.60%) in T650 treatment. After comparison with bacterial community structure among 20 soil samples, the bacterial community structure had commonalities and differences at the order level. The commonality was that *Sphingomonadales*(4.96–11.31%) *and Sphingobacteriales*(4.60–9.97%) had higher average relative abundance in T0, T20, T30, T500, and T650 treatments, *Gp4*(norank_*Acidobacteria*_Gp4) in T0, T30, and T500 treatments, *Gp6*(norank_*Acidobacteria*_Gp6) in T20, T30, and T500 treatments; The differences was that *Rhizobiales*(8.38%) had higher average relative abundance in T0 treatment, *Burkholderiales*(20.01%) and *Bacteroidales*(10.27%) in T650 treatment.

**Figure 17:**
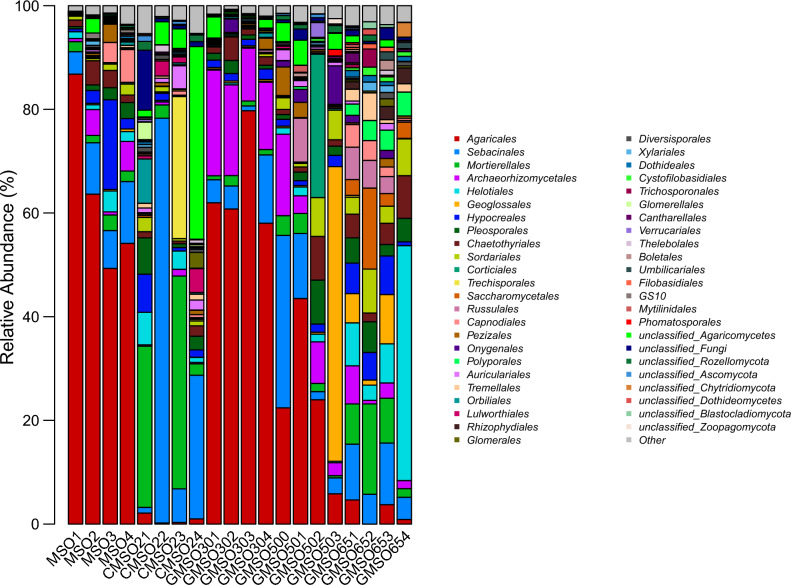
The relative abundance of fungal communities in 20 soil samples at the order level.

At the order level, the relative abundance of fungal communities in 20 soil samples was shown in Fig. 17. *Agaricales*(63.48%), *Sebacinales*(8.36%), and *Hypocreales*(5.59%) had higher average relative abundance in T0 treatment; *Sebacinales*(28.34%), *Mortierellales*(19.24%), and *Trechisporales*(6.94%) in T20 treatment; *Agaricales*(65.13%), *Archaeorhizomycetales*(15.27%), and *Sebacinales*(5.74%) in T30 treatment; *Agaricales*(23.93%), *Geoglossales*(14.46%), *Sebacinales*(12.62%), *Archaeorhizomycetales*(7.42%), and *Corticiales*(6.99%) in T500 treatment; *Helotiales*(16.03%), *Mortierellales*(8.88%), *Sebacinales* (8.17%), *Saccharomycetales* (6.06%), and *Sordariales* (5.54%) in T650 treatment. After comparison with fungal community structure among 20 soil samples, the fungal community structure had commonalities and differences at the order level. The commonality was that *Agaricales* had much higher average relative abundance in T0, T30, and T500 treatments(23.93–65.13%), *Sebacinales* in T0, T20, T30, T500 and T650 treatments(5.74–28.34%), *Archaeorhizomycetales* in T30 and T500 treatments(7.42–15.27%), *Mortierellales* in T20 and T650 treatments(8.88–19.24%). The differences was that *Hypocreales*(5.59%) only had higher average relative abundance in T0 treatment, *Trechisporales*(6.94%) in T20 treatment, *Geoglossales*(14.46%) and *Corticiales*(6.99%) in T500 treatment, *Helotiales*(16.03%), *Saccharomycetales* (6.06%) and *Sordariales* (5.54%) in T650 treatment.

### The community structure of bacteria and fungi at the family level

**Figure 18:**
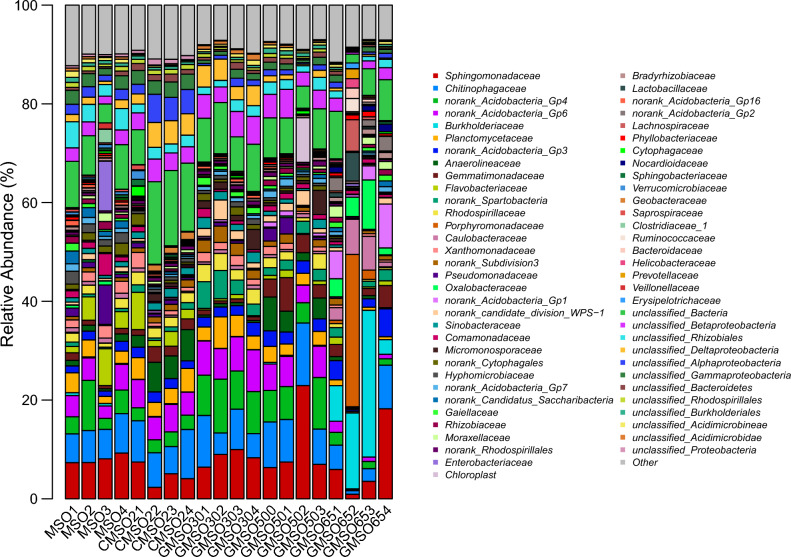
The relative abundance of bacterial communities in 20 soil samples at the family level.

At the family level, the relative abundance of bacterial communities in 20 soil samples was shown in Fig. 18. *Sphingomonadaceae*(8.01%), *Chitinophagaceae*(6.57%), and *Gp4*(norank_*Acidobacteria*_Gp4)(5.16%) had higher average relative abundance in T0 treatment; *Chitinophagaceae*(7.71%), *Gp6*(norank_*Acidobacteria*_Gp6)(5.14%), *Anaerolineaceae*(4.83%), and *Sphingomonadaceae*(4.74%) in T20 treatment; *Gp4*(norank_*Acidobacteria*_Gp4)(8.83%), *Sphingomonadaceae*(8.43%), *Gp6* (norank_*Acidobacteria*_Gp6)(7.12%), and *Chitinophagaceae*(6.97%) in T30 treatment; *Sphingomonadaceae*(10.93%), *Chitinophagaceae*(9.42%) *Gp4*(norank_*Acidobacteria*_Gp4)(6.90%), and *Gp6*(norank_*Acidobacteria*_Gp6)(5.32%) in T500 treatment; *Burkholderiaceae*(13.81%), *Porphyromonadaceae*(8.40%), *Sphingomonadaceae*(7.16%), *Oxalobacteraceae*(4.75%), *Gp1*(norank_*Acidobacteria*_Gp1)(4.47%), *Caulobacterales*(4.37%), and *Chitinophagaceae*(4.26%) in T650 treatment*.* After comparison with bacterial community structure among 20 soil samples, the bacterial community structure had commonalities and differences at the family level. The commonality was that *Sphingomonadales*(4.74–8.43%) *and Chitinophagaceae*(4.26–7.71%) had higher average relative abundance in all 20 soil samples, *Gp4*(norank_*Acidobacteria*_Gp4)(5.16–8.83%) in T0, T30, T500 treatments, *Gp6*(norank_*Acidobacteria*_Gp6)(5.14–7.12%) in T20, T30, T500 treatments; The differences was that *Anaerolineaceae*(4.83%) only had higher average relative abundance in T20 treatment, *Burkholderiaceae*(13.81%), *Porphyromonadaceae*(8.40%), *Oxalobacteraceae*(4.75%), *Gp1*(norank_*Acidobacteria*_Gp1)(4.47%), and *Caulobacterales*(4.37%) in T650 treatment.

**Figure 19:**
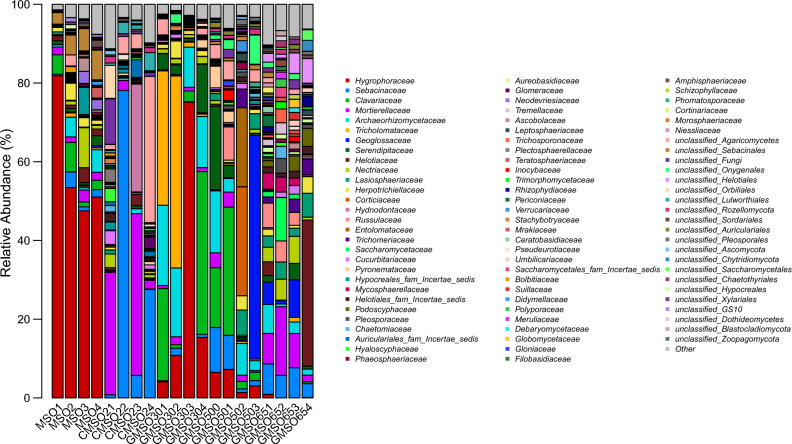
The relative abundance of fungal communities in 20 soil samples at the family level.

At the family level, the relative abundance of fungal communities in 20 soil samples was shown in Fig. 19. *Hygrophoraceae*(58.45%) and *Clavariaceae*(4.03%) had higher average relative abundance in T0 treatment; *Sebacinaceae*(28.02%), *Mortierellaceae*(19.24%), and *Hygrophoraceae*(6.94%) in T20 treatment; *Hygrophoraceae*(26.34%), *Tricholomataceae*(21.18%), *Clavariaceae*(17.10%), *Archaeorhizomycetace*(15.27%), and *Serendipitaceae*(4.85%) in T30 treatment; *Geoglossaceae*(14.37%), *Clavariaceae*(12.94%), *Archaeorhizomycetace*(7.42%), *Corticiaceae*(6.99%), *Serendipitaceae*(6.30%), *Sebacinaceae*(5.58%), *Entolomataceae*(5.14%), and *Hygrophoraceae*(4.55%) in T500 treatment; *Helotiaceae*(10.16%), *Mortierellaceae*(8.88%), *Sebacinaceae*(6.17%), and *Nectriaceae*(4.15%) in T650 treatment, After comparison with fungal community structure among 20 soil samples, the fungal community structure had commonalities and differences at the family level. The commonality was that *Hygrophoraceae* had very higher average relative abundance in T0, T20, T30, and T500 treatment(4.55–58.45%), especially in T0 treatment, the relative abundance reached 58.45%, *Clavariaceae*(4.03–17.10%) in T0, T30, T500 treatments, *Archaeorhizomycetace*(7.42–15.27%) in T30, T500 treatments, *Sebacinaceae*(5.58–28.02%) in T20, T500, T650 treatments, *Mortierellales*(8.88–19.24%) in T20 and T650 treatments, *Serendipitaceae*(4.85–6.30%) in T30 and T500 treatments; The differences was that *Clavariaceae*(4.03%) only had higher average relative abundance in T0 treatment, *Tricholomataceae*(21.18%) in T30 treatment, *Geoglossaceae* (14.37%), *Corticiaceae* (6.99%), and *Entolomataceae*(5.14%) in T500 treatment, *Helotiaceae*(10.16%) and *Nectriaceae* (4.15%) in T650 treatment.

### The community structure of bacteria and fungi at the genus level

**Figure 20:**
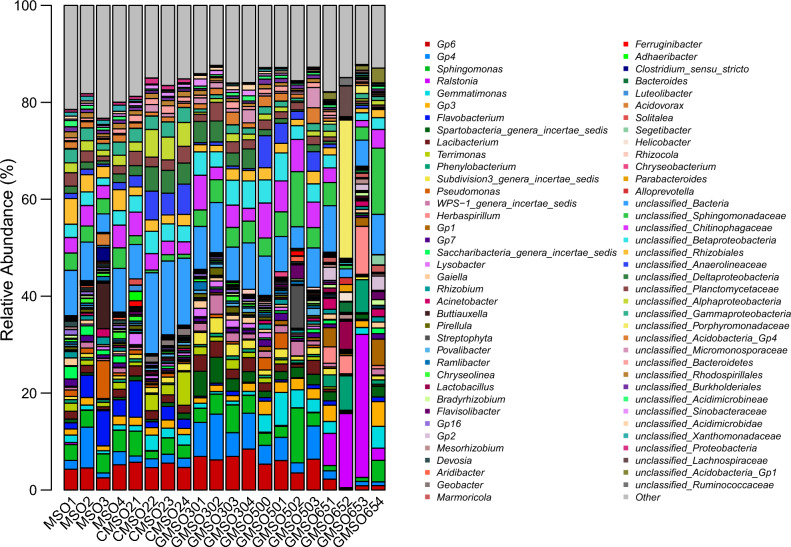
The relative abundance of bacterial communities in 20 soil samples at the genus level.

At the genus level, the relative abundance of bacterial communities in 20 soil samples was shown in Fig. 20. *Gp6*(*norank_Acidobacteria*_Gp6)(4.14%), *Flavobacterium*(4.12%), *Sphingomonas*(3.77%), *Gp4*(*norank_Acidobacteria*_Gp4)(3.49%) had higher average relative abundance in T0 treatment; *Gp6*(*norank_Acidobacteria*_Gp6)(5.14%), *Flavobacterium*(3.37%), *Sphingomonas*(3.30%), *Gemmatimonas*(3.19%) in T20 treatment; *Gp4*(*norank_Acidobacteria*_Gp4)(7.20%), *Gp6*(7.12%), *Sphingomonas*(4.11%), *Spartobacteria_genera_incertae_sedis*(3.82%) in T30 treatment; *Gp6*(*norank_Acidobacteria*_Gp6)(5.32%), *Sphingomonas*(4.70%), *Gp4*(4.40%), and *Gemmatimonas*(4.21%) in T500 treatment; *Ralstonia*(13.49%), *Herbaspirillum* (4.43%), and *Phenylobacterium*(4.33%) in T650 treatment. After comparison with bacterial community structure among 20 soil samples, the bacterial community structure had commonalities and differences at the genus level. The commonality was that *Sphingomonas*(3.30–4.70%) had higher average relative abundance in T0, T20, T30, and T500 treatments, *Gp6*(*norank_Acidobacteria*_Gp6)(4.14–7.12%) in T0, T20, T30, T500 treatments, *Flavobacterium*(3.37–4.12%) in T0 and T20 treatments, *Gp4*(*norank_Acidobacteria*_Gp4)(3.49–7.20%) in T0, T30, and T500 treatments, *Gemmatimonas*(3.19–4.21%) in T20 and T500 treatments; The differences was that *Spartobacteria_genera_incertae_sedis*(3.82%) only had higher average relative abundance in T30 treatment, *Ralstonia* (13.49%), *Herbaspirillum*(4.43%), and *Phenylobacterium*(4.33%) in T650 treatment.

**Figure 21:**
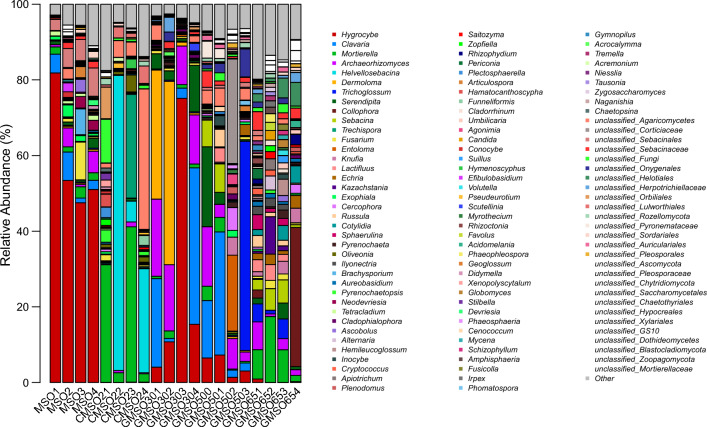
The relative abundance of fungal communities in 20 soil samples at the genus level.

At the genus level, the relative abundance of fungal communities in 20 soil sample was shown in Fig. 21. *Hygrocybe*(58.45%), *Clavaria*(4.03%), *Archaeorhizomyces*(2.98%), and *Fusarium*(2.88%) had higher average relative abundance in T0 treatment; *Helvellosebacina*(27.85%), *Mortierella*(19.24%), *Trechispora*(6.94%) in T20 treatment; *Hygrocybe*(26.34%), *Dermoloma*(21.04%), *Clavaria*(17.10%), *Archaeorhizomyces* (15.27%), and *Serendipita* (4.85%) in T30 treatment; *Trichoglossum*(13.9%), *Clavaria*(12.94%), *Archaeorhizomyces*(7.42%), *Serendipita*(6.30%), *Entoloma*(5.06%), *Hygrocybe*(4.55%), *Sebacina*(3.76%) in T500 treatment; *Collophora*(9.74%), *Mortierella*(8.88%), *Sebacina*(3.84%), *Archaeorhizomyces*(3.14%) in T650 treatment. After comparison with fungal community structure among 20 soil samples, the fungal community structure had commonalities and differences at the genus level. The commonality was that *Archaeorhizomyces*(2.98–15.27%) had very higher average relative abundance in T0, T30, T500, and T650 treatments, *Hygrocybe*(4.55–58.45%) in T0, T30, and T500 treatments, *Clavaria*(4.03–17.10%) in T0, T30, T500 treatments, *Mortierella*(8.88–19.24%) in T20 and T650 treatments; The differences was that *Fusarium*(2.88%) only had higher average relative abundance in T0 treatment, *Helvellosebacina*(27.85%) and *Trechispora* (6.94%) in T20 treatment, *Dermoloma*(21.04%) and *Serendipita*(4.85%) in T30 treatment, *Trichoglossum*(13.9%), *Serendipita*(6.30%), *Entoloma*(5.06%), and *Sebacina*(3.76%) in T500 treatment, *Collophora*(9.74%), *Sebacina*(3.84%) in T650 treatment.

### The relationship between soil environment factors with bacterial and fungal abundance

**Figure 22:**
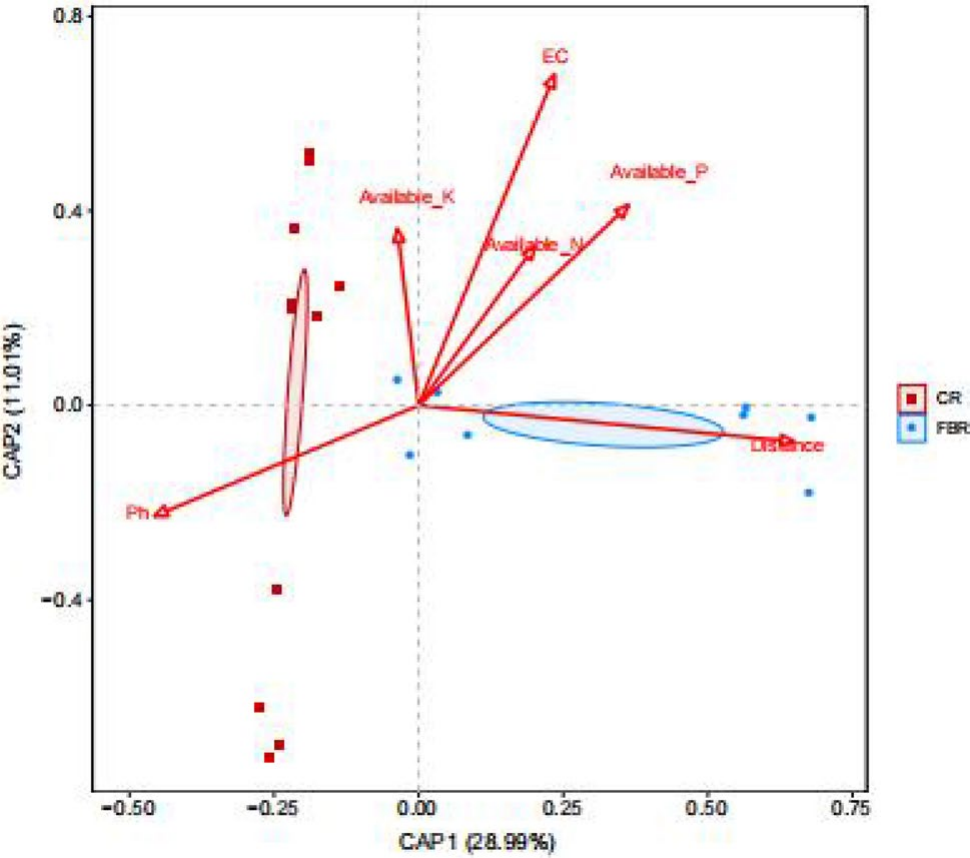
Redundancy analysis (db-RDA) between soil environment factors and bacterial abundance of CR and FBR group at OTU level.

The relationship between soil environment factors and bacterial community abundance of CR and FBR group soil samples was shown in the Fig. 22. The results showed that among 6 soil environmental factors, distance, pH and EC had very important effects on the distribution of bacterial communities, In particular, the distance had the greatest effect, indicating that mercury in the soil played a dominant role in the distribution of bacterial communities. The bacterial community abundance of FBR group soil samples was more affected by distance than that in CR group soil samples.

**Figure 23:**
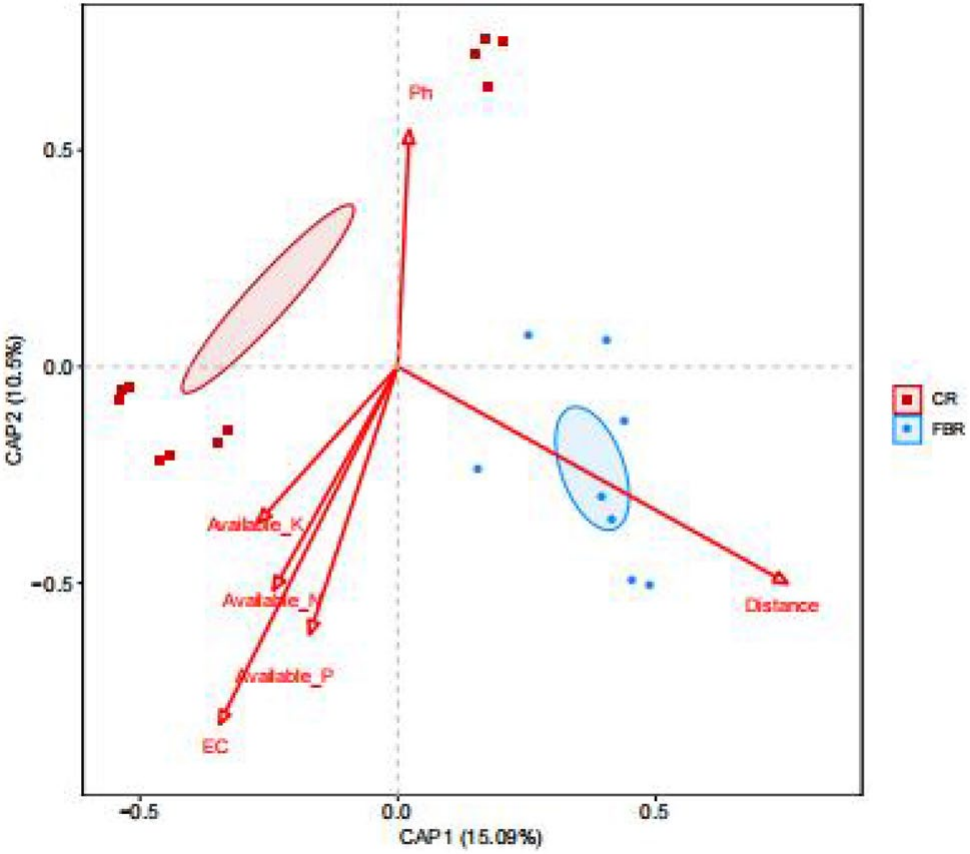
Redundancy analysis (db-RDA) between soil environment factors and fungal abundance of CR and FBR group at OTU level.

The relationship between soil environment factors and fungal community abundance of CR and FBR group soil samples was shown in the Fig. 23. The results showed that among the 6 soil environmental factors, distance, EC and pH had very important effects on the distribution of fungal communities, In particular, the distance had the greatest effect, indicating that mercury in the soil played a dominant role in the distribution of fungal communities. The fungal community abundance of FBR group soil samples was more affected by distance than that of CR group soil samples. While the fungal community abundance of CR group soil samples was more affected by pH, and EC than that of FBR group soil samples.

## Discussion

Mercury is one of the most common heavy metal pollutants in the environment. Soil mercury pollution is often closely related to the mining of surrounding mercury-related metal mines and the smelting of mercury-related metals. Once soil is polluted by mercury, it will be transferred to plant roots, stems, leaves and other parts through plant enrichment. When the soil mercury content continues to rise, it will seriously affect the normal growth and development of plants^18,19^. Meanwhile, it will have adverse effects on the soil ecosystem, especially on soil microorganisms and soil animals^20^. Previous studies have confirmed that the concentration of Cd, Zn, Pb and Cu in soil had a strong correlation with soil microbial biomass^21^, and different microbial groups had different tolerance to different heavy metals^22^. The reason why heavy metals had adverse effects on microbial diversity could be that high concentration heavy metals had a certain restrictive effect on microbial cell metabolism and other functions, which ultimately led to a decrease in microbial diversity^23^.

In this work, the results of bacterial and fungal community composition in soils with different mercury contents at different distances from mercury mining areas showed that the number of bacterial and fungal communities in 20 soil samples increased significantly from the order level to the genus level. The number of uniquely distributed bacterial and fungal communities to each soil sample of the 20 soil samples showed a significant increase trend from the order level to the genus level. This result showed that even in a certain mercury stress environment, the number of bacterial and fungal communities in the soil increased with the decline of the grade level, which was not significantly affected by soil mercury and other factors. Meanwhile, the alpha diversity of bacterial and fungal communities were analyzed by using reads, OTUs, Shannon index and Simpson index (showed in Tables 2 and 3), our results indicated that there were large differences in the diversity of bacterial and fungal communities among 4 replicates soil samples, from T0 to T650 treatments, the diversity of bacterial communities decreased gradually, however, the diversity of fungal communities increased gradually. This results indicated that in the outdoor natural environment, the diversity distribution of bacterial and fungal communities at different points within the same range was affected by the internal factors of the soil. The diversity of bacterial and fungal communities was affected by many factors, resulting in the difference in the number of uniquely distributed bacterial and fungal communities to individual soil samples^24^. It also indicated that there was a certain heterogeneity in the soil around the mercury mining area. Therefore, it can be speculated that mercury press will lead to a decline in soil microbial diversity to a certain extent, but the relationship between the two was not a simple linear relationship, also affected by other factors within the soil. Meanwhile, the results on beta diversity analysis of bacterial and fungal communities showed that there were some differences between bacterial and fungal communities, the similarity of bacterial communities from far to near was T20, T0, T500, T30, and T650 treatment soil samples, the similarity of fungal communities from near to far was T0, T30, T20, T650, and T500 treatment soil samples. The similarity of bacterial communities and fungal communities in different soil samples was significantly different, and the main reason for this result was related to the internal heterogeneity of soil.

The results of this study showed that the number of bacterial communities in T0 and T20 treatments was much larger than that in T30, T500, and T650 treatments at order, family and genus level. Also lying in fungal communities. That is, the number of bacteria and fungal communities decreased with decline of mercury concentration at order, family and genus level. This result was inconsistent with the research results of Shan and coworkers^25^. This could be caused by the difference of mercury concentration or experimental environment. Previous studies belonged to the artificial simulation laboratory soil environment. This study was the natural soil environment of mercury mining area, various bacterial and fungal communities reached an equilibrium state after a long period of comprehensive influence of various factors inside the soil. The real existence of these bacterial and fungal communities was a good adaptation to mercury-containing soil environment for a long time. The reason for this result could be related to the soils of different types. Maliszewska and coworkers^26^found that the compounds of Cu and Hg showed the strongest inhibitory effect on microbial proliferation, and the order of toxicity of HgCl_2_ to microorganisms was bacteria > fungi > actinomycetes. The results showed that the inhibitory effect of mercury on microbial proliferation was fluctuated, and the inhibition of microorganisms decreased with the prolongation of culture time^26^. In this work, the soil around the mercury mining area had been subjected to high concentration mercury pollution stress for a long time. The increase in the number of bacterial and fungal communities under higher concentration mercury environment could be the best evidence that the inhibition of bacterial and fungal communities decreased with time.

The structure of bacterial, fungal and communities of actinomycetes in soil was easily affected by environmental variables around the soil, such as nutrient cycling and heavy metal concentration changed^27,28^. In this work, the effects of mercury concentration on the structure of bacterial and fungal communities at the order, family and genus levels were analyzed. The results indicated that mercury had a certain effect on the structure of bacterial and fungal communities, and the tolerance of different bacterial and fungal communities to mercury pollution was also very different, which was consistent with the results of Prasad^29^. Our results indicated that the dominant bacterial communities in T0–T650 treatments were different at order, family and genus level. At the order level, *Sphingomonadales* and *Sphingobacteriales* were the dominant bacterial communities, the relative abundance remained at about 10%; at the family level, for *Sphingomonadaceae* and *Chitinophagaceae*; at the genus level, for *Gp6*(norank_Acidobacteria_Gp6), *Sphingomonas*,*Gp4* (norank_Acidobacteria_Gp4). Similar results were also found in fungal communities, at the order level, *Agaricales* and *Sebacinales* were the dominant fungal communities; at the family level, for *Sebacinaceae* and *Hygrophoraceae*; at the genus level, for *Hygrocybe* and *Sebacina*. Regardless of the classification level, bacterial and fungal communities with higher relative abundance were the performance of long-term adaptation to higher mercury soil environment, indicating that they had strong tolerance to heavy mercury stress. This was similar to the conclusion of Feris^24^ and Gillan et al.^30^. Meantime, at order, family and genus level, the dominant bacterial and fungal communities in different positions of mercury mining area were quite different, which was closely related to the heterogeneity of many factors in the internal environment of soil. The relationship between soil environment factors and bacterial and fungal community abundance was analyzed by using db-RDA method at OTU level, the results indicated that distance(Hg^2+^concentration), EC, and pH three soil factors had greater effects on the relative abundance of bacterial and fungal communities than available N, P, K. Among distance(Hg^2+^concentration), EC, and pH, distance(Hg^2+^concentration) had the greatest effects on the relative abundance of bacterial and fungal communities. This results fully demonstrated that these dominant bacterial and fungal communities had much stronger resistance to mercury pollution stress.

## Conclusion

From the perspective of the effects of mercury stress on bacterial and fungal communities at different distances from mercury mining areas, Based on the experimental results, we could infer the following conclusions. 1. Under the same classification, the diversity of bacterial and fungal communities in the proximal soil was much higher than that in the distal soil; 2. At the same classification level, among five different distance soils sample, there were both the same dominant bacterial and fungal communities and different dominant bacterial and fungal communities; 3. The structure and distribution of bacterial and fungal communities were highly correlated with soil environment factors, and the mercury content in soil had more important effect on the structure and distribution of bacterial and fungal communities.

## Data Availability

The raw sequence data reported in this paper have been deposited in the Genome Sequence Archive (Genomics, Proteomics & Bioinformatics 2021) in National Genomics Data Center (Nucleic Acids Res 2022), China National Center for Bioinformation/Beijing Institute of Genomics, Chinese Academy of Sciences (GSA: CRA011955) that are publicly accessible at https://ngdc.cncb.ac.cn/gsa. Please cite the following required publications: [1] The Genome Sequence Archive Family: Toward Explosive Data Growth and Diverse Data Types. Genomics, Proteomics & Bioinformatics 2021, 19(4):578–583. https://doi.org/10.1016/j.gpb.2021.08.001 [PMID = 34400360]. [2] Database Resources of the National Genomics Data Center, China National Center for Bioinformation in 2022. Nucleic Acids Res 2022, 50(D1):D27–D38. https://doi.org/10.1093/nar/gkab951 [PMID = 34718731].
